# Weight-Bearing Versus Non-Weight-Bearing After Ankle Fracture: A Systematic Review and Meta-Analysis of Patient-Reported Outcome

**DOI:** 10.3390/life15020314

**Published:** 2025-02-18

**Authors:** Rafael Llombart-Blanco, Gonzalo Mariscal, Ibrahim Khalil, Violeta Cordón, María Benlloch, Carlos Barrios, Rafael Llombart-Ais

**Affiliations:** 1 Orthopedic Surgery Department, University Clinic of Navarra, 31008 Pamplona, Spain; rllombartb@gmail.com; 2 Institute for Research on Musculoskeletal Disorders, School of Medicine, Valencia Catholic University, 46001 Valencia, Spain; gonzalo.mariscal@mail.ucv.es (G.M.); violetacordon@icloud.com (V.C.); carlos.barrios@ucv.es (C.B.); rllombartivcot@gmail.com (R.L.-A.); 3 Faculty of Medicine, Alexandria University, Alexandria 21526, Egypt; ibrahim.ibrahim1908@alexmed.edu.eg; 4 Department of Basic Biomedical Sciences, Catholic University of Valencia, 46001 Valencia, Spain; 5 Traumacenter, Casa de Salud Hospital, 46021 Valencia, Spain

**Keywords:** ankle fracture, weight bear, patient-reported outcomes, immobilization, meta-analysis

## Abstract

Background: Ankle fractures commonly affect mobility and quality of life. Although unstable fractures typically require surgery, post-treatment protocols vary widely, including both surgical and conservative approaches. This systematic review and meta-analysis evaluated the effects of early weight-bearing after ankle fracture treatment on functional outcomes and quality of life. Methods: Following the PRISMA guidelines and the PICOS strategy, we performed a meta-analysis across multiple databases (PubMed, Embase, Scopus, and Cochrane Library). Analysis was conducted using Review Manager 5.4, calculating the mean and standard mean differences with 95% confidence intervals (CIs). Results: Eleven studies (*n* = 939) showed favorable outcomes with weight-bearing. Significant functional improvements were observed at 6 weeks (MD 7.88, 95% CI 3.14–12.61), 3 months (MD 5.79, 95% CI 4.41–7.17), and 12 months (MD 4.74, 95% CI 3.01–6.46). RAND scores favored the weight-bearing group at 6 weeks (SMD 0.48, 95% CI 0.26–0.70) and 12 months (SMD 0.31, 95% CI 0.07–0.55), with no significant differences at 3 months (SMD 0.18, 95% CI −0.10–0.46). Conclusion: The outcomes obtained indicated statistically significant differences in favor of the early weight-bearing group regarding ankle function and quality of life.

## 1. Introduction

Ankle fractures are frequently encountered injuries that significantly impair movement and affect the overall quality of life of individuals across all age groups. They are common among adults, with an annual incidence ranging from 122 to 187 per 100,000 people [1,2]. This rate is expected to escalate due to an increasing number of individuals participating in athletic activities [3] and an increase in the elderly population, which is estimated to account for 16% of the population by 2050 [4]. Despite the difficulty in classifying ankle fractures, they can be divided into two categories based on the severity of accompanying soft tissue injury [2]. Stable ankle fractures typically involve a solitary fibular break while maintaining the integrity and stability of the ankle joint. These fractures can often be managed non-surgically and constitute approximately half of all cases of ankle fractures [5]. Conversely, unstable ankle fractures, which include fracture dislocations, isolated malleolar fractures with talar shift, and bimalleolar and trimalleolar fractures, usually require surgical intervention to ensure proper healing and functionality [6].

While proper anatomical joint reconstruction is crucial for ankle fracture recovery, post-fracture management protocols remain controversial [7]. The predominant rehabilitation protocol advocates immobilization using a cast or orthosis for six weeks, during which weight bearing is strictly prohibited [8]. An alternative strategy promotes primary wound healing in orthoses. Followed by early mobilization and tolerating weight-bearing, the rationale behind the extended immobilization protocol is primarily safety-driven, premised on the belief that early mobilization increases the risk of fracture displacement or other related complications [7]. Evidence supporting this view points to higher complication rates associated with weight-bearing [9]. However, contrasting studies have demonstrated improved outcomes or no significant differences when implementing weight-bearing [7].

The concept of early weight-bearing after ankle fractures has received growing attention in orthopedic practice, mirroring the shift that has been seen in the postoperative management of other types of fractures [6,10]. This is based on the biological principle that controlled mechanical loading has the potential to stimulate bone healing through mechanotransduction, increase blood flow, and prevent muscular atrophy [11,12]. Physiological benefits that result from early weight-bearing involve the acceleration of osteoblast activity, callus formation, and remodeling of fracture sites [13,14]. Early weight-bearing also helps prevent complications of immobility, such as contractures, muscle wasting, and osteoporosis [15,16]. This biological rationale, along with advances in surgical fixation techniques and an improved understanding of fracture healing [17,18], has resulted in increased interest in early weight-bearing protocols for ankle fractures.

The lack of consensus regarding follow-up care for ankle fractures presents clinical challenges. This meta-analysis evaluated treatment approaches by combining multiple studies with substantial statistical evidence.

A recent meta-analysis examining the effects of weight-bearing post-surgical treatment of ankle fractures focused primarily on functional recovery of the ankle [10]. However, there is a compelling need to expand this research to include additional studies and to consider other critical factors, such as quality of life metrics, which were not considered.

This meta-analysis evaluated the benefits of weight-bearing versus non-weight-bearing after ankle fracture treatment, focusing on functional recovery and quality of life outcomes. Our goal is to provide evidence-based recommendations for optimal rehabilitation protocols.

## 2. Materials and Methods

### 2.1. Eligibility Criteria

The systematic review and meta-analysis adhered to a registered protocol with PROSPERO (CRD42024525196) and followed the PRISMA guidelines for transparency and reproducibility [19]. Using the PICOS framework, we selected studies that met the specific criteria. The study population included adults aged >18 years with ankle fractures, either surgically treated for unstable fractures or conservatively managed for stable, non-displaced fractures. To address potential heterogeneity, subgroup analyses were planned to account for differences in fracture stability and associated weight-bearing (WB) protocols. The intervention group consisted of patients instructed on weight-bearing, with weight-bearing explicitly defined in terms of timing (such as immediate, early, or delayed) and magnitude (partial or full weight-bearing). The comparison group adhered to standard non-weight-bearing protocols. The primary outcomes focused on patient-reported measures of ankle function and quality of life, assessed using validated functional tools, such as the American Orthopaedic Foot and Ankle Society (AOFAS) Ankle-Hindfoot Score or equivalent scales. We included only comparative studies, such as randomized clinical trials or cohort studies, to maintain methodological rigor. Duplicates, case reports, letters to editors, non-comparative studies, pediatric populations, trial protocols, and studies with incomplete data were excluded, ensuring that only unique and comprehensive studies relevant to adult populations were analyzed.

### 2.2. Information Sources

We systematically searched PubMed, EMBASE, SCOPUS, and Cochrane Library without language or publication status restrictions. In addition to searching the databases, we also reviewed the reference lists of the included studies and relevant reviews to identify other potentially eligible papers.

### 2.3. Search Methods for Identification of Studies

The search methods combined controlled vocabulary and keywords such as “weight-bearing” and “ankle fracture”. Two independent reviewers screened all the retrieved titles and abstracts. The full texts of potentially relevant reports were evaluated according to pre-specified eligibility criteria. Any discrepancies between reviewers during the initial screening or full-text evaluation stages were resolved through discussion until a consensus was reached.

### 2.4. Data Extraction and Data Items

Two reviewers performed data extraction independently, with disagreements resolved through discussion. Extracted data included study characteristics, demographics, funding sources, and group descriptions. Ankle function was assessed using the Olerud and Molander Ankle Score (OMAS), ranging from 0 to 100 [20], the AOFAS, which ranges from 0 to 100 [21], and the Baird-Jackson scale, with scores from 0 to 100 [17], with higher scores in these three scales indicating better function. The RAND-36 score was used to measure health-related quality of life from 0 to 100 points, with a higher score indicating better quality of life [22]. The outcomes were compared at 6 weeks, 3 months, and 12 months follow-up after the surgery or after a week of conservative treatment if the patients did not undergo surgery [18,23].

### 2.5. Assessment of Risk of Bias in Included Studies

The Cochrane risk-of-bias tool evaluated six domains in randomized controlled trials: random sequence generation, allocation concealment, blinding of participants and personnel, blinding of outcome assessment, incomplete outcome data, and selective reporting. Each domain received a rating of low, high, or unclear risk.

The Methodological Index for Non-Randomized Studies (MINORS) criteria were applied [24]. For comparative studies, a maximum score of 24 was possible. Scores between 0 and 6 were considered very low quality, 7–10 as low quality, 11–15 as fair quality, and 16 or higher as high quality [24].

### 2.6. Assessment of Quality of Evidence

The GRADE (Grading of Recommendations Assessment, Development, and Evaluation) system was used. The GRADE system was applied to each outcome included in our meta-analysis [25], considering factors such as study design, result consistency, estimation precision, potential biases, and clinical relevance of the findings.

### 2.7. Assessment of Results

Meta-analyses were conducted using RevMan 5.4.1 statistical software. Continuous outcomes were evaluated using mean differences (MDs) or standardized mean differences (SMDs) with 95% confidence intervals (CIs). The SF-12 and SF-36, which are versions of the RAND scale assessing the quality of life, were combined using SMDs for a more straightforward interpretation. For dichotomous outcomes, we used the odds ratio along with the corresponding 95% confidence interval (CI). We employed the fixed effects model for the pooled analysis; however, if substantial heterogeneity (*p* < 0.1, I^2^ > 50%) was identified, we shifted to the random effects model (DerSimonian Liard method). This model considers the variability within and between studies by allocating a greater standard error to the pooled estimate. Thus, it accounts for disparate effect sizes by favoring studies with small sample sizes over those with large ones. Consequently, it is necessary to account for these potential discrepancies in the estimates. We assessed heterogeneity using the Cochrane Q test (chi-squared test) and measured it using Higgins and Thompson I-squared, which quantifies the proportion of variability in effect estimates attributed to heterogeneity rather than chance. We considered a significant chi-square test at an alpha level of less than 0.1, and we considered substantial heterogeneity if the I-square was >50%. To investigate the source of heterogeneity, we performed a sensitivity analysis on significant heterogeneity in several scenarios, excluding one study at a time, and reran the analysis [26].

### 2.8. Risk of Bias Across the Studies

Publication bias was assessed using funnel plots in Review Manager 5.4, examining the symmetry of study effects against standard errors. While asymmetry suggests potential bias, formal statistical tests (Egger’s, Begg’s) were unavailable in the software.

## 3. Results

### 3.1. Study Selection

**Figure 1:**
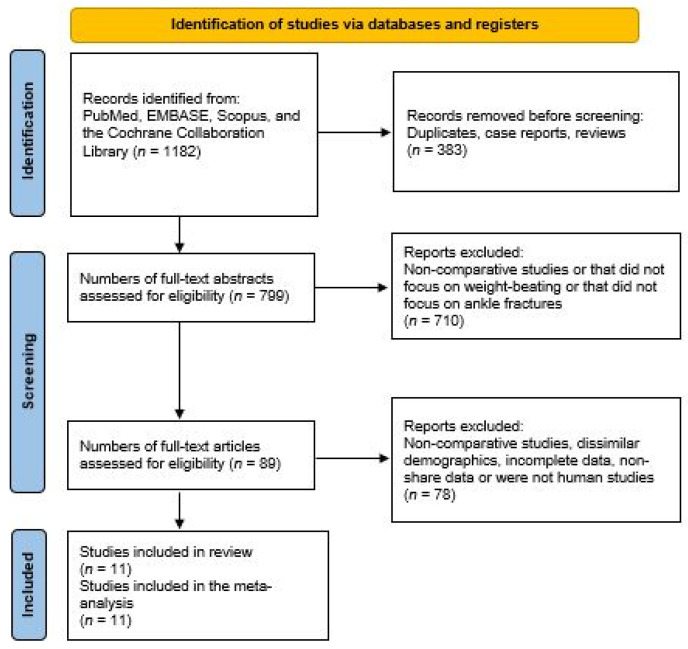
Study selection flow diagram (PRISMA).

The literature search initially identified 1182 articles across various databases. After removing duplicates, case reports, and prior reviews, 383 studies were excluded, leaving 799 articles. A detailed review of the abstracts was conducted to refine this selection further, focusing on eliminating either non-comparative studies, those that did not address the comparison between weight-bearing and non-weight-bearing treatments, or those that were unrelated to ankle fractures. This process narrowed down the field to 89 pertinent studies. Subsequent scrutiny of the full-text articles led to the exclusion of an additional 78 studies. The reasons for exclusion included a lack of comparative analysis, demographic discrepancies, incomplete datasets, data that were not shared, or studies that did not involve human subjects. Following this rigorous selection process, 11 studies met all inclusion criteria and were included in the meta-analysis. These studies are referenced in Figure 1 and cited in the references [2,3,7,15,18,23,27,28,29,30,31].

### 3.2. Study Characteristics

Table 1 presents the baseline characteristics of the studies included in this meta-analysis. The analysis included 11 studies involving 939 participants, with 486 in the weight-bearing group and 453 in the non-weight-bearing group. The observation period ranged from 3 to 24 months. In the weight-bearing group, the average age varied from 37.8 to 83.1 years, while in the non-weight-bearing group, it ranged from 37.8 to 82.6 years. Details regarding the number of women, conflicts of interest, and funding are presented in Table 1. Additionally, a detailed description of the experimental and control groups is provided in Supplementary Table S1.

### 3.3. Risk of Bias

**Figure 2:**
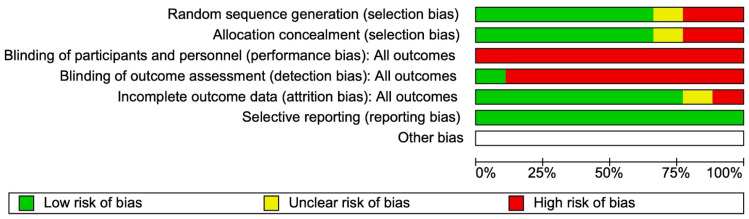
Risk of bias graph.

Nine studies were randomized clinical trials, and two were comparative studies. Figure 2 and Supplementary Figure S1 show the quality and risk of bias assessments for randomized studies using the Cochrane Review Manager risk of bias tool. Several studies were judged as having some concerns for their methodological quality, with an unclear or high risk of bias found in the following domains: blinding of participants and personnel, the nine studies did not adequately blind participants or personnel from group assignment because it was not possible because the patients had to know their treatment to follow it; blinding of outcome assessment, eight studies did not have blinding of outcome assessment; three studies did not have adequate random sequence generation, three studies showed a selection bias, and two studies had too many report losses to follow-up, which was registered as incomplete outcome data.

Two comparative studies were evaluated using the MINORS tool and were deemed high quality (18 and 19 points) (Supplementary Table S2).

### 3.4. Outcomes

**Figure 3:**
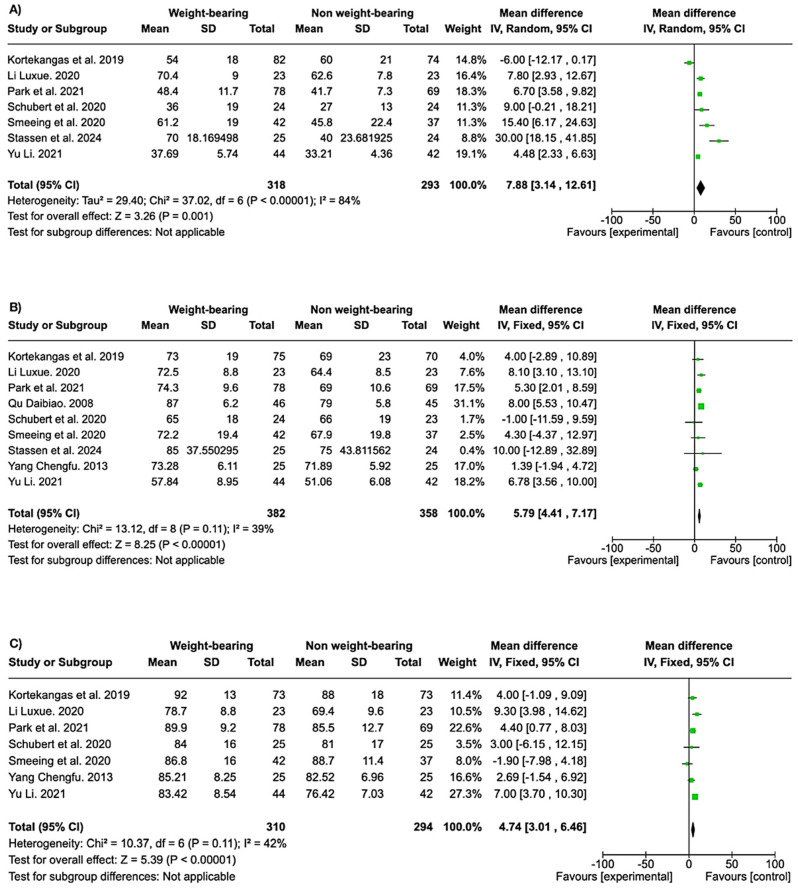
Forest plots showing significant differences in ankle function at 6 weeks (**A**), 3 months (**B**), and 6 months (**C**) in favor of the weight-bearing group.

Regarding ankle function, weight-bearing showed favorable results globally. Significant differences occurred at six weeks (MD 7.88, 95% CI 3.14 to 12.61; participants = 611; studies = 7; I^2^ = 84%) (Figure 3A). At the three-month follow-up, weight-bearing showed significantly favorable results (MD 5.79, 95% CI 4.41 to 7.17; participants = 740; studies = 9; I^2^ = 39%) (Figure 3B). At 12 months, there were significant differences in favor of the weight-bearing group (MD 4.74, 95% CI 3.01 to 6.46; participants = 604; studies = 7; I^2^ = 42%) (Figure 3C).

**Figure 4:**
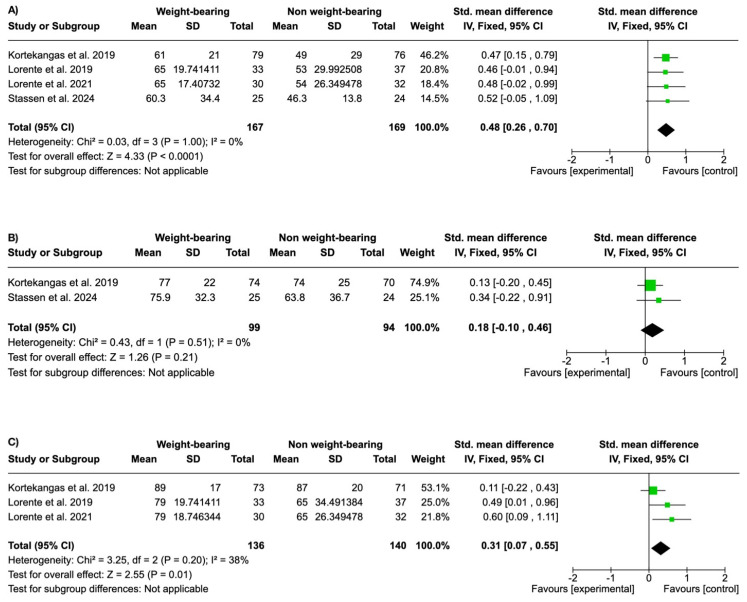
Forest plots showing significant differences in the RAND score at 6 weeks in favor of the weight-bearing group (**A**), no significant differences at 3 months (**B**), and significant differences in the RAND score at 12 months in favor of the weight-bearing group (**C**).

The RAND score showed significantly more favorable results in the weight-bearing group at 6 weeks (SMD 0.48, 95% CI 0.26 to 0.70; participants = 336; studies = 4; I^2^ = 0%) (Figure 4A). There were no significant differences at 3 months (SMD 0.18, 95% CI −0.10 to 0.46; participants = 193; studies = 2; I^2^ = 0%) (Figure 4B), but there were significant differences in favor of the weight-bearing group at the 12-month follow-up (SMD 0.31, 95% CI 0.07 to 0.55; participants = 276; studies = 3; I^2^ = 38%) (Figure 4C).

### 3.5. Additional Analysis

Subgroup analyses were performed for the following groups: stable Weber B nonoperative fractures (same fracture classification, comparing weight-bearing vs. non-weight-bearing) and unstable ankle fracture operations (same fracture classification, comparing weight-bearing vs. non-weight-bearing) at three clinically relevant time points (6 weeks, 3 months, and 6 months).

For Stable Weber B Non-Operative fractures (two studies, 205 participants; I^2^ = 96%), weight bearing showed significantly better ankle function compared to non-weight bearing [MD = 11.63, 95% CI (−23.64, 46.90)] at six weeks (Supplementary Figure S2). For the unstable ankle fractures operative group (five studies, 413 participants; I^2^ = 43%), weight bearing also demonstrated significantly better ankle function versus non-weight bearing [MD = 6.81, 95% CI (4.29, 9.44)] at six weeks. The test for subgroup differences (χ^2^ = 0.07, df = 1, *p* = 0.79) indicated no statistically significant differences between fracture types. At 3 months, for stable Weber B non-operative fractures (two studies, 194 participants; I^2^ = 0%), weight bearing showed no significant difference in ankle function compared to non-weight bearing [MD = 4.67, 95% CI (−1.86, 11.19)] (Supplementary Figure S3). For the unstable ankle fractures operative group (seven studies, 546 participants; I^2^ = 53%), weight bearing showed significantly better ankle function compared to non-weight bearing [MD = 5.85, 95% CI (4.44, 7.26)]. The test for subgroup differences (χ^2^ = 0.12, df = 1, *p* = 0.73) indicated no statistically significant differences between fracture types. And at 6 months, for stable Weber B non-operative fractures (one study, 146 participants; I^2^ not applicable), weight bearing showed no significant difference in ankle function versus non-weight bearing [MD = 4.00, 95% CI (−1.09, 9.09)] (Supplementary Figure S4). For the unstable ankle fractures operative group (six studies, 458 participants; I^2^ = 51%), weight bearing maintained significantly better ankle function compared to non-weight bearing [MD = 4.83, 95% CI (3.00, 6.66)]. The test for subgroup differences (χ^2^ = 0.09, df = 1, *p* = 0.76) indicated no statistically significant differences between fracture types.

**Figure 5:**
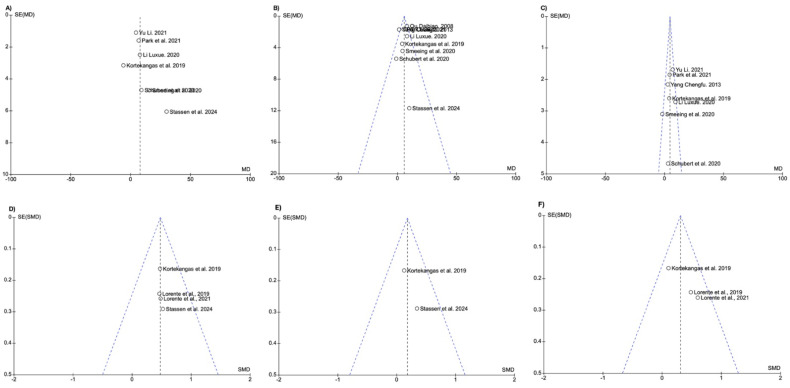
On the top of the figure, funnel plots show heterogeneity in ankle function at 6 weeks follow-up (**A**) and no publication bias in ankle function at 3 months (**B**) and 6 months follow-up (**C**). On the bottom of the figure, funnel plots demonstrate no publication bias in the RAND score at 6 weeks (**D**) and 3 months (**E**) and 6 months follow-up (**F**).

Upon visual inspection of funnel plots, heterogeneity was observed in the plot for ankle function at the 6-week follow-up, suggesting potential publication bias for those outcomes (Figure 5A).

### 3.6. Grade Summary

The GRADE assessment revealed different levels of certainty for each outcome. For ankle function, the evidence was low at 6 weeks owing to high heterogeneity (I^2^ = 84%) and potential publication bias. At 3 and 12 months, the evidence was moderate, as heterogeneity was lower, and no publication bias was detected.

For the RAND score, the evidence was moderate at 6 weeks and 12 months owing to consistent results and low heterogeneity. However, at three months, the evidence was low because there were few studies, and no significant differences were found.

The risk of bias was due to the lack of blinding in most studies, although the two comparative studies were of high quality. Despite these issues, the results suggest that weight-bearing protocols improve recovery after ankle fracture. (Supplementary Table S3).

## 4. Discussion

In this meta-analysis, we compared early weight-bearing with non-weight-bearing in terms of ankle function and quality of life in patients over 18 years of age with ankle fractures surgically or conservatively treated in the short and long term. With regard to ankle function, weight-bearing showed favorable results globally. The RAND score assessing life quality showed significantly more favorable results in the weight-bearing group at 6 weeks; there were no significant differences at 3 months, but there were significant differences in favor of the weight-bearing group at the 12-month follow-up.

The standard postoperative management for ankle fractures typically involves immobilization and a below-the-knee cast for six weeks, based on the understanding that it takes approximately six weeks for bones to fuse sufficiently to withstand the stresses of weight-bearing activities [2].

However, weight-bearing after ankle fracture surgery has been associated with theoretical risks, such as displacement of fixed fractures, implant failure, and loss of reduction [3,7]. Studies have also shown an increased rate of complications, including problems with the surgical wound, when early weight-bearing is implemented [7]. Additionally, early mobilization may hinder fracture healing, leading to a greater need for interventions and increased overall procedural costs [18]. Despite these concerns, a cadaver study found that under the stress of weight-bearing activities, fractures shift by less than 1 mm [32]. This outcome is supported by biomechanical analyses that show no implant failure or loss of reduction when weight-bearing is implemented for surgically treated ankle fractures [13]. Furthermore, postoperative immobilization has several disadvantages, such as soft tissue atrophy, stiffness, loss of strength [18], and a higher risk of osteoporosis [7]. Therefore, weight-bearing seems to be a suitable protocol for ankle fractures. Many studies have stated that weight-bearing can be safely started two weeks after surgery [3]. Weight-bearing could also offer several benefits to patients’ recovery. It may help restore the range of motion of the injured joint more quickly and enable faster rehabilitation [18]. A Cochrane review also suggested that weight-bearing could reduce activity limitation and pain [17]. Regarding quality of life, weight-bearing could result in an earlier return to preinjury activities [3]. and other works [15]. The patients’ satisfaction with being allowed to walk early should also be considered [3].

In this study, we focused on ankle function, measured using the OMAS, and the patient’s quality of life, measured using the RAND scale. These variables allowed us to evaluate the success of the treatments, focusing on the patients’ re-establishment more realistically and practically than physiological and biomechanical studies. Ultimately, the patients’ most important outcomes were function and quality of life. The OMAS is a 0–100 ordinal rating scale with higher scores indicating better function [33], and the RAND-36 is a widely used questionnaire for evaluating health-related quality of life [22]. We have only incorporated in the statistical analysis the physical component of this scale to be able to compare the results with the quality of life results that some studies included in our meta-analysis in older versions of the same system, such as the Short Form-12 Health Survey (SF-12), which established only a score for each patient and did not separate physical and mental components. Both scales’ reliability, validity, and measurement properties have demonstrated favorable outcomes [22,33].

If we look at the follow-up time, we see that the improvement in the OMAS is maintained both in the short and long term (Figure 3). In the RAND score, there were no significant differences at 6 weeks, but there were in the long term (Figure 4), and in the end, the long term is what matters in the patient’s recovery. Therefore, it would be interesting to have follow-up periods of more than 12 months in future studies.

We inspected the outcomes by applying the Minimal Clinically Important Difference (MCID) of the RAND scale and OMAS, the most used ankle function scale in the included studies. We could not perform a formal analysis because the included studies did not report the number of patients that reached the MCID in each group; however, we performed an indirect analysis. Regarding OMAS, the MCID ranges from 10.5 to 15.0 [34]. Let us now examine our results in this context. There seem to be no clinically relevant differences between the weight-bearing and non-weight-bearing groups, although there were statistically significant differences. The same occurs with the RAND MCID, which ranges from 4.9 to 5.21, with no apparent clinically relevant differences between weight-bearing and non-weight-bearing patients [35]. Considering each study individually, it can be seen that Smeeing et al. [15] and Stassen et al. [27]. had outcomes showing clinically relevant differences in favor of early weight bearing for the OMAS at 3 weeks follow-up. This fact supports the idea that early weight bearing may have benefits for the patient in terms of ankle function in the short term.

When examining studies that included surgically treated patients versus conservative treatment, there were only two conservative studies [18,23] versus nine surgical studies. The patients in the studies by Lorente et al. [18,23] were older, and nonoperative treatment was chosen [18]. Upon visual inspection of the funnel plots, no differences were expected in the analysis of this subgroup of old, non-surgically treated patients compared to other studies. Robust sensitivity analyses could not be conducted because of the small sample size.

Weight-bearing has been proposed as a post-surgical treatment for other pathologies, such as pelvic fractures. Murena et al. stated that even when postoperative guidelines regarding restricted or limited weight-bearing are clearly outlined, many studies indicate that patients often struggle to comply. This noncompliance frequently results in patients exceeding the recommended weight-bearing limits [14]. Weight-bearing could be an easier option and ensure compliance with treatment. Furthermore, some authors consider that weight-bearing can trigger micro-movements between the fragments of the fracture, which may promote the healing process [11]; however, to date, there is limited clinical evidence available to support the use of weight-bearing in the treatment of pelvic and acetabular fractures [14].

However, the potential benefits of initiating weight-bearing early after lower limb fractures have been acknowledged for decades. Garden advocated early weight-bearing following surgical fixation of femoral neck fractures. Da Costa and Kumar found that allowing weight-bearing sooner than 6 weeks after conservative treatment of tibial shaft fractures resulted in faster bone union without an increase in complications [36]. Evidence supports weight-bearing after surgical fixation of various lower limb fractures, such as femoral neck fractures, femoral shaft fractures, ankle fractures, and Schatzker I–III plateau fractures [12]. Weight-bearing has also been recommended for tibial shaft fractures treated with external fixation, plates, and screws. Bhanushali et al. evaluated the effect of early versus delayed weight-bearing after intramedullary nailing of tibial shaft fractures. They concluded that weight-bearing may lead to quicker healing and a lower overall rate of complications in patients who do not have obvious risk factors [12].

Similarly to these outcomes, the results of our study on weight-bearing after ankle fractures indicate significant differences in ankle function and quality of life. We have not thoroughly investigated the complications associated with each post-surgical management option; however, further research in this area is necessary to establish clear guidelines for the management of ankle fractures. Although our study focused on evaluating functional and quality of life outcomes, when examining the complications reported by the original studies, including fracture displacement, non-union, reoperations, and surgical site infections, among others, it was observed that none showed significant differences between the weight-bearing and non-weight-bearing groups [7,15,18,23].

Our large-scale analysis yielded strong supportive evidence for early weight-bearing in ankle fractures according to detailed subgroup comparisons based on fracture classifications. For stable Weber B nonoperative fractures, weight-bearing demonstrated significantly improved ankle function at 6 weeks (MD = 11.63, 95% CI [23.64, 46.90]), with sustained but nonsignificant improvements at 3 and 6 months. Similar compelling evidence was found in unstable ankle fractures treated operatively with weight-bearing, providing functional significance at each time point: at 6 weeks [MD = 6.81, 95% CI (4.29, 9.44)], 3 months [MD = 5.85, 95% CI (4.44, 7.26)], and 6 months [MD = 4.83, 95% CI (3.00, 6.66)]. The results, with respect to specific fracture classifications, provide definitive evidence in favor of the benefits of weight-bearing protocols while taking into consideration the diverse characteristics and demands of each type of fracture. These benefits move upstream with well-known biological mechanisms of fracture healing, such that controlled mechanical loading encourages osteoblast activity and blood flow at a fracture site [11]. The early functional benefits, especially those seen at 6 weeks post-surgery, suggest that mechanical stimulation by means of weight-bearing can potentially accelerate healing by stimulating early callus formation and inhibiting the negative aspects of immobilization [13]. Mere homogenous results (e.g., when we do not differentiate between fractures in place or the different needs for weight-bearing between a neck of femur fracture and a patella fracture) should not be regarded as concrete proof for the generalized benefits of weight-bearing strategies.

### Limitations

Our study had several limitations. Initially, analyzing outcomes for more than 12 months of follow-up was impossible. The included studies did not blind the patients to the type of treatment they should follow, which may lead to a high risk of bias in this domain. Moreover, the absence of formal statistical assessments for publication bias, such as Egger’s and Begg’s tests in Review Manager 5.4, restricts our ability to evaluate potential publication bias thoroughly. It is essential to highlight those studies like Lorente et al. that investigated the impact of weight-bearing on non-surgically treated ankle fractures, introducing complexity to the result interpretation. Finally, our analysis was limited by the scarcity of articles exploring confounding variables. In future research, it would be beneficial to present the variables after statistical adjustment.

A major limitation of this analysis is that we did not have long-term follow-up data regarding post-traumatic ankle arthritis. Although our study was able to demonstrate enhanced functional outcomes within the first year of early weight-bearing, the occurrence of post-traumatic arthritis continues to be one of the most important long-term complications of ankle fractures and is observed 3–5 years after injury. This endpoint may be more clinically important than functional scores in the short term. This uncertainty led to exploratory studies with long-term follow-up to resolve whether post-traumatic arthritis was induced by early weight-bearing protocols. For future research, a longer follow-up (3–5 years following injury) will also be needed to determine the potential protective effects of weight-bearing protocols on post-traumatic ankle arthritis, including radiographic and symptomatic assessments. This outcome could prove to be more clinically meaningful than functional scores measured over the short term when considering optimal treatment protocols.

## 5. Conclusions

In conclusion, this study conducted a rigorous analysis of the weight-bearing versus non-weight-bearing management of ankle fractures. The results demonstrated statistically significant improvements in ankle function and quality of life in the weight-bearing group. These differences were notable and were sustained throughout the 12-month follow-up period.

Future research should focus on providing data adjusted for confounding variables to enhance the reliability of the findings. Additionally, it is crucial to report whether individual patients reach the MCID, offering a deeper insight into the clinical relevance of early weight-bearing on a per-patient basis.

These findings underscore the potential benefits of weight-bearing protocols for the management of ankle fractures. Implementing such protocols could lead to quicker functional recovery and improved patient quality of life, which may reduce the healthcare costs associated with extended rehabilitation periods and delayed return to work. Therefore, healthcare providers should consider these benefits when designing postoperative care plans for patients with ankle fractures.

## Figures and Tables

**Table 1:** Baseline characteristics of the eleven included studies.

Study	Region	Period	Follow-Up (Months)	*n* Weight-Bearing/Control	Age Weight-Bearing/Control	Female Weight-Bearing/Control	Conflict of Interest (Yes/No)	Funding (Yes/No)
Lorente et al. 2020 [18]	Spain	2014 to 2018	24	33/37	82.9/82.4	18/21	No	NR
Lorente et al. 2021 [23]	Spain	2015 to 2018	24	30/32	83.1/82.6	15/19	No	No
Kortekangas et al. 2019 [2]	Finland	2012 to 2016	12	83/84	46/45	40/38	Yes	Yes
Park et al. 2021 [3]	Republic of Korea	2014 to 2017	12	95/99	42.7/43.1	41/39	No	NR
Schubert et al. 2020 [7]	Australia	2012 to 2016	6	24/21	46/42	12/9	No	No
Smeeing et al. 2020 [15]	The Netherlands	2013 to 2016	12	42/37	37.8/37.8	20/14	No	No
Stassen et al. 2024 [27]	The Netherlands	2018 to 2019	3	25/24	52/51	8/17	No	No
Luxue et al. 2020 [28]	China	2016 to 2019	6	23/23	47.4/45.9	8/6	NR	NR
Yang et al. 2013 [29]	China	1999 to 2010	6	25/25	46.9/45.2	7/6	NR	NR
Qu et al. 2008 [30]	China	NR	NR	62/29	55.1/55.8	28/17	NR	NR
Li et al. 2021 [31]	China	2018 to 2020	6	44/42	39.18/38.75	19/20	No	NR

NR: not reported.

## Data Availability

All data relevant to the study are included in the article or uploaded as online supplemental information. Data may be available upon reasonable request from the corresponding author.

## Supplementary Materials

The following supporting information can be downloaded at https://www.mdpi.com/article/10.3390/life15020314/s1, Figure S1: Risk of bias summary; Figure S2: Forest plot of subgroup analyses regarding the following groups were performed: stable Weber B non-operative fractures and unstable ankle fractures operative at six weeks; Figure S3: Subgroup analyses regarding the following groups were performed: stable Weber B non-operative fractures and unstable ankle fractures operative at three months; Figure S4: Subgroup analyses regarding the following groups were performed: stable Weber B non-operative fractures and unstable ankle fractures operative at six months; Table S1: Description of the intervention and control group; Table S2: Assessment of the quality of studies through Methodological Index for Non-Randomized Studies (MINORS); Table S3: GRADE summary.

## Abbreviations

The following abbreviations are used in this manuscript:

GRADE	Grading of Recommendations Assessment, Development, and Evaluation
AOFAS	American Orthopaedic Foot and Ankle Society
OMAS	Olerud and Molander Ankle Score
MINORS	Methodological Index for Non-Randomized Studies
MCID	Minimal Clinically Important Difference
