# Supplementary material for: Weight-Bearing Versus Non-Weight-Bearing After Ankle Fracture: A Systematic Review and Meta-Analysis of Patient-Reported Outcome
Source: Life (Basel). 2025 Feb 18;15(2):314. doi: 10.3390/life15020314 (PMC11857458; doi:10.3390/life15020314)
Supplement: Supplementary file 1 [file life-15-00314-s001.zip › Supplementary Table S1.docx]

**Supplementary Table S1.** Description of the intervention and control group

| **Study** | **Weight-bearing group** | **Control group** |
| --- | --- | --- |
| **Lorente et al. 2020 [18]** | The weight-bearing activities consisted of walking with support from two crutches or a walker based on tolerance to balance and pain. | The patients were immobilized in a closed plaster without support orthosis for six to eight weeks. |
| **Lorente et al. 2021 [23]** | The patients were taught how to load their limb adequately using a plastic brace or crutches. Correct loading was defined as ability of the patient to completely step the foot on the ground. This fact was verified by the orthopedic surgeon during the follow up period in the clinical room. To exclude secondary displacements and ensure adequate fracture healing, control X-rays were performed every 2weeks until the sixth or eight week. Both groups used the same type of plaster cast, which was maintained for 6–8 weeks, depending on the extent of consolidation viewed on the follow-up X-rays. | The patients used a plaster cast , which was maintained for 6–8 weeks, depending on the extent of consolidation viewed on the follow-up X-rays. |
| **Kortekangas et al. 2019 [2]** | A trained plaster technician applied a standard below-the-knee cast (3M Scotchcast; St Paul, MN). The cast was applied from the tuberosity of the tibia to the base of the toes and was lined and padded. Participants received guidance from a physiotherapist on walking with crutches. Weightbearing was permitted immediately after the application of the cast. Participants received written and verbal instructions on to how to cope with the ankle fracture and support. The cast was removed at the three week follow-up visit. The patients were instructed to continue weightbearing as tolerated with no need for further bracing or support. | A trained plaster technician applied a standard below-the-knee cast (3M Scotchcast; St Paul, MN). The cast was applied from the tuberosity of the tibia to the base of the toes and was lined and padded. Participants received guidance from a physiotherapist on walking with crutches. Weightbearing was permitted immediately after the application of the cast. Participants received written and verbal instructions on to how to cope with the ankle fracture and support.The cast was removed at the three week follow- up visit. The patients received a new, below-the-knee cast. |
| **Park et al. 2021 [3]** | The Patients were allowed to bear weight, as tolerated, in a removable walking cast at 2 weeks postoperatively for the next 4 weeks. Daily active and passive range of motion exercises of the ankle joint were encouraged at 2 weeks postoperatively, after wound healing. Six weeks after the operation, the cast or splint was removed and more active exercise was recommended. | The patients were maintained in an NWB state in a removable splint with crutches until 6 weeks postoperatively. Range of motion exercises of the ankle joint were started at 2 weeks postoperatively. Six weeks after the operation, the cast or splint was removed in both groups and more active exercise was recommended. |
| **Schubert et al. 2020 [7]** | The patients were allowed to weight bear as tolerated within the CAM boot from 2 weeks postoperatively. Daily active and passive range-of-motion exercises of the ankle and subtalar joints without the brace were encouraged. | The patients were not allowed to weight bear on the affected leg for a total of 6 weeks. These patients were allowed range-of-motion exercises 3 times a day out of their CAM boot. Daily active and passive range-of-motion exercises of the ankle and subtalar joints without the brace were encouraged. |
| **Smeeing et al. 2020 [15]** | The treatment included a pressure dressing in the first 24 h postoperatively. After the first 24 h, weight-bearing was allowed as tolerated by the patient. The patients consulted a physical therapist postoperatively to learn exercises and received advice on how to start mobilizing. | The treatment included a pressure dressing in the first 24 h postoperatively. After 6 weeks, weight-bearing was allowed. The patients consulted a physical therapist postoperatively to learn exercises and received advice on how to start mobilizing. |
| **Stassen et al. 2024 [27]** | The patients were fitted a walking boot (Rebound® Air Walker, Össur, Reykjavik, Iceland) and were instructed on permissive weightbearing and use of the walking boot. Instructions included the removal of the walking boot when not weightbearing to mobilize the affected ankle. The patients were prescribed daily use of Dalteparin 5.000 IE for the duration of the intervention. After six weeks, the walking boot was removed and patients were instructed to functionally use the ankle again. There was no transition from walking boot to a brace, as patients were instructed permissive weightbearing. | A cast below the knee was applied by a trained plaster technician. The cast was applied from a few centimeters below the tuberositas tibiae to the base of the toes. The ankle was fixed in a plantigrade position. Patients were specifically instructed not to bear weight on the affected limb. If any cast-related complaints occurred during treatment, a new cast was fitted. The patients were prescribed daily use of Dalteparin 5.000 IE for the duration of the intervention. After six weeks, the cast was removed and patients were instructed to functionally use the ankle again. There was no transition from cast to a brace, as patients were instructed permissive weightbearing. |
| **Luxue et al. 2020 [28]** | The patients underwent open reduction and internal fixation. They did not receive plaster external fixation. 24 hours after surgery, the patients were instructed to perform active contraction of the lower limb muscles. On the third day after surgery, the patients were instructed to perform active flexion and extension of the ankle joint in bed. In the first week after surgery, the patients were instructed to walk on the ground with crutches, but the affected side did not touch the ground. Crutches were allowed in the second week after surgery, and the patients were instructed to walk on the toes of the affected side as far as they could tolerate. In the fourth week after surgery, the weight was gradually increased according to the recovery of the affected side. Three times a day; in the sixth week after surgery, the affected ankle joint was reviewed for anteroposterior and lateral X-rays. If callus growth appeared at the fracture end, the affected side could walk with the whole foot with crutches, and if there was no callus growth, the patients were instructed to continue walking on the toes with crutches. | The patients underwent open reduction and internal fixation. After surgery, the affected side was fixed with plaster for 2 weeks. Before the plaster was removed, active flexion and extension training was performed on the metatarsophalangeal joint, and active and passive search training was performed on the lower limb muscles. Two weeks after surgery, the plaster was removed, and the patients were asked to perform active and passive flexion and extension activities of the affected ankle joint on the bed. In the fourth week after surgery, the patients walked on the ground with the help of crutches, but the affected side could not touch the ground. In the sixth week after surgery, the affected ankle joint was re-examined for anteroposterior and lateral X-rays, and the affected side could walk on tiptoe. |
| **Yang et al. 2013 [29]** | The patients underwent surgery and were afterwards treated with oral Chinese medicine. They began to bear weight early after surgery. | The patients underwent surgery and were afterwards treated with oral Chinese medicine. They started to bear weight late after the surgery. |
| **Qu et al. 2008 [30]** | Weight-bearing 2 weeks after surgery. | Weight-bearing 6 weeks after surgery. |
| **Li et al. 2021 [31]** | The patients received rehabilitative trainings for joint activity, muscle strength and proprioception. While starting from the 4th week postoperatively, these patients received partial weight-bearing training as well. The patients held the parallel rod with both hands, and put the mass measuring instrument under feet to experience the loading weight they can tolerate. The patient’s initial loading weight was 25% of his body mass, which ranged from 10 to 20 kg, and then increased progressively. The patients looked straight and walked with crutches in a three-point manner, and started to stand or walk with full weight 13 weeks after surgery. | The patients received rehabilitative trainings for joint activity, muscle strength and proprioception.  According to fracture degree, the patients performed the partial weight-bearing standing or walking in the 13th to 24th weeks after surgery, and gradually increased the exercise intensity to full weight-bearing. |
