# Supplementary material for: Weight-Bearing Versus Non-Weight-Bearing After Ankle Fracture: A Systematic Review and Meta-Analysis of Patient-Reported Outcome
Source: Life (Basel). 2025 Feb 18;15(2):314. doi: 10.3390/life15020314 (PMC11857458; doi:10.3390/life15020314)
Supplement: Supplementary file 1 [file life-15-00314-s001.zip › Supplementary Figure S3.docx]

**Supplementary Figure S3**

**
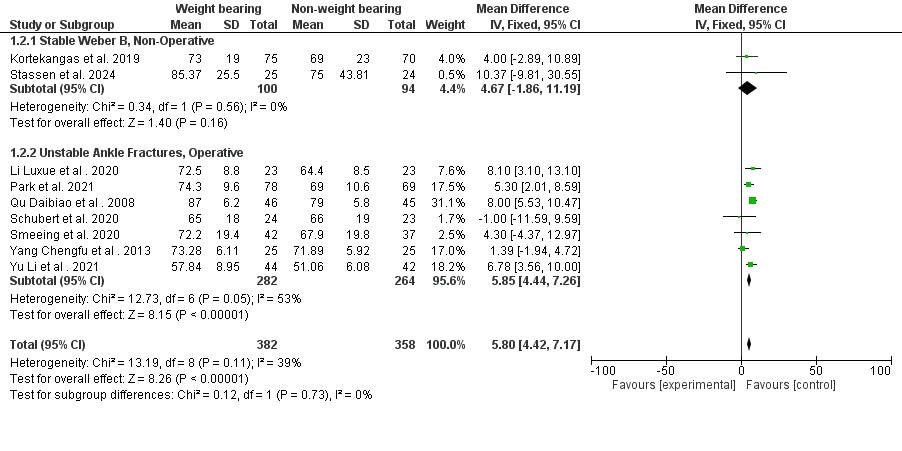
**

**Supplementary Figure S3.** Forest Plot of Subgroup analyses regarding the following groups were performed: stable Weber B Non-Operative fractures and unstable Ankle Fractures Operative at three months. In the fixed effects model subgroup analyses, for Stable Weber B Non-Operative fractures, weight bearing showed no significant difference in ankle function compared to non-weight bearing [MD = 4.67, 95% CI (-1.86, 11.19)]. For Unstable Ankle Fractures Operative group, weight bearing showed significantly better ankle function compared to non-weight bearing [MD = 5.85, 95% CI (4.44, 7.26)].
