# Supplementary material for: Weight-Bearing Versus Non-Weight-Bearing After Ankle Fracture: A Systematic Review and Meta-Analysis of Patient-Reported Outcome
Source: Life (Basel). 2025 Feb 18;15(2):314. doi: 10.3390/life15020314 (PMC11857458; doi:10.3390/life15020314)
Supplement: Supplementary file 1 [file life-15-00314-s001.zip › Supplementary Table S2.docx]

| **Study** | **Clearly stated aim** | **Consecutive patients** | **Prospective collection data** | **Endpoints** | **Assessment endpoint** | **Follow-up period** | **Loss less than 5%** | **Study size** | **Adequate control group** | **Contemporary group** | **Baseline control** | **Statistical analyses** | **MINORS** |
| --- | --- | --- | --- | --- | --- | --- | --- | --- | --- | --- | --- | --- | --- |
| **Lorente et al. 2019 [18]** | 2 | 1 | 2 | 2 | 0 | 2 | 1 | 0 | 2 | 2 | 2 | 2 | 18 |
| **Lorente et al. 2021 [23]** | 2 | 2 | 2 | 2 | 0 | 2 | 1 | 0 | 2 | 2 | 2 | 2 | 19 |

**Supplementary Table S2.** Assessment of the quality of studies through Methodological Index for Non-Randomized Studies (MINORS).
