# Supplementary figures and images for: Weight-Bearing Versus Non-Weight-Bearing After Ankle Fracture: A Systematic Review and Meta-Analysis of Patient-Reported Outcome
Source: Life (Basel). 2025 Feb 18;15(2):314. doi: 10.3390/life15020314 (PMC11857458; doi:10.3390/life15020314)

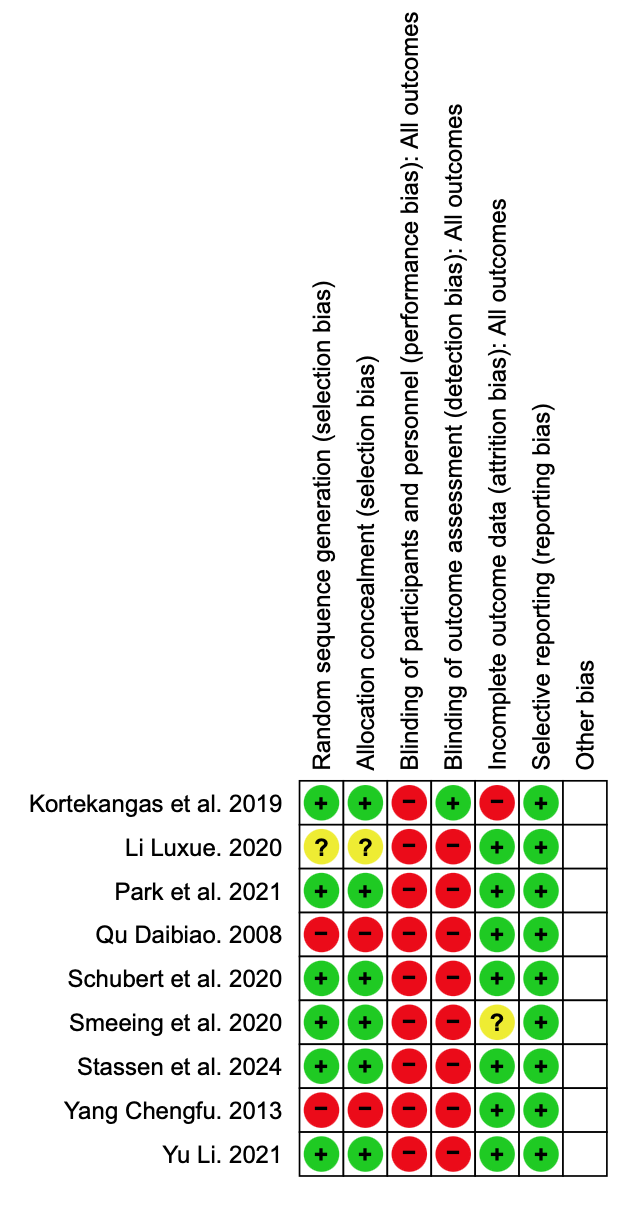

Supplement: Supplementary file 1 [file life-15-00314-s001.zip › Supplementary Figure S1.png]
