# Supplementary material for: Weight-Bearing Versus Non-Weight-Bearing After Ankle Fracture: A Systematic Review and Meta-Analysis of Patient-Reported Outcome
Source: Life (Basel). 2025 Feb 18;15(2):314. doi: 10.3390/life15020314 (PMC11857458; doi:10.3390/life15020314)
Supplement: Supplementary file 1 [file life-15-00314-s001.zip › Supplementary Table S3.doc]

**Supplementary Table S3** GRADE summary

| **Outcome** | **No. of Studies** | **Study Design** | **Risk of Bias** | **Inconsistency** | **Indirectness** | **Imprecision** | **Publication Bias** | **Number of Participants** | **Effect (95% CI)** | **Certainty of Evidence** |
| --- | --- | --- | --- | --- | --- | --- | --- | --- | --- | --- |
| Ankle Function (6 weeks) | 7 | RCTs & Comparative | Some concerns | High (I² = 84%) | None | None | Suspected bias | 611 | MD 7.88 (3.14 to 12.61) | Low |
| Ankle Function (3 months) | 9 | RCTs & Comparative | Some concerns | Low (I² = 39%) | None | None | None | 740 | MD 5.79 (4.41 to 7.17) | Moderate |
| Ankle Function (12 months) | 7 | RCTs & Comparative | Some concerns | Low (I² = 42%) | None | None | None | 604 | MD 4.74 (3.01 to 6.46) | Moderate |
| RAND Score (6 weeks) | 4 | RCTs & Comparative | Some concerns | Low (I² = 0%) | None | None | None | 336 | SMD 0.48 (0.26 to 0.70) | Moderate |
| RAND Score (3 months) | 2 | RCTs & Comparative | Some concerns | Low (I² = 0%) | None | None | None | 193 | SMD 0.18 (-0.10 to 0.46) | Low |
| RAND Score (12 months) | 3 | RCTs & Comparative | Some concerns | Low (I² = 38%) | None | None | None | 276 | SMD 0.31 (0.07 to 0.55) | Mode |
